# Nano Titanium Dioxide Particles Promote Allergic Sensitization and Lung Inflammation in Mice

**DOI:** 10.1111/j.1742-7843.2009.00473.x

**Published:** 2010-02

**Authors:** Søren T Larsen, Martin Roursgaard, Keld A Jensen, Gunnar D Nielsen

**Affiliations:** National Research Centre for the Working EnvironmentCopenhagen, Denmark

## Abstract

The purpose of this study was to investigate whether photocatalytic TiO_2_ nanoparticles have adjuvant effect, when administered in combination with ovalbumin (OVA) in mice. Mice were immunized via intraperitoneal injections of OVA, OVA + TiO_2_ or OVA + Al(OH)_3_ and challenged with aerosols of OVA. At the end of the study, serum was analysed for content of OVA-specific IgE, IgG1 and IgG2a antibodies, and the bronchoalveolar lavage fluid (BALF) was analysed for content of inflammatory cells and levels of interleukin (IL)-4, IL-5, IL-10 and interferon-γ. The TiO_2_ particles promoted a Th2 dominant immune response with high levels of OVA-specific IgE and IgG1 in serum and influx of eosinophils, neutrophils and lymphocytes in BALF. The TiO_2_ particles induced a significantly higher level of OVA-specific IgE than the standard adjuvant Al(OH)_3_. However, the two substances were comparable regarding the level of eosinophilic inflammation and interleukins present in BALF.

Epidemiological as well as laboratory studies have provided evidence that exposure to ambient particulate matter is associated with different health effects, including airway inflammation [1,2], allergic sensitization [3–7] and exacerbation of asthma [8,9]. Thus, diesel exhaust particles are able to increase sensitization against allergens both in animals and humans [6,7,10]. Recently man-made nanomaterials, including single-walled and multi-walled carbon nanotubes as well as latex nanoparticles, have been shown to promote allergic sensitization [11], allergic lung inflammation [12,13] and airway fibrosis [14].

In contrast to e.g. diesel exhaust particles and carbon nanotubes, titanium dioxide (TiO_2_) particles are generally accepted to be low-toxic to humans as well as animals [15–17]. The presumed low toxicity of TiO_2_ is reflected in the wide use of this compound, for example as a pigment in paints, food, medicine and sunscreens [18–20]. Micrometer-sized TiO_2_ particles showed no adjuvant effect when administered to mice in combination with ovalbumin (OVA) [21].

For many substances, including TiO_2_, a reduction of particle size is associated with increased pro-inflammatory properties [22] and oxidative damage in human bronchial epithelial cell line [23]. With the development of nanotechnology and manufacturing of products containing nanoparticles, knowledge of the role of particle size on the toxicity is of increasing importance. Only one previous study [21] has investigated the effect of nano-sized TiO_2_ on the immune response to simultaneously administered allergen, but very fine particles of widely different composition possessed adjuvant effect in a mouse model using OVA as model allergen [24]. Also, adjuvant effect of particles on allergic sensitization has been shown to increase with decreasing particle size [21,25].

Some of the newly marketed ‘nano’ household spray products with self-cleaning properties contain nano-sized photocatalytic TiO_2_, why human airway exposures to such particles may be increasing. Similarly, paints are deliberately added nanoparticulate TiO_2_ to obtain improved properties and self-cleaning effects. Numerous other products may also contain TiO_2_ nanoparticles. The present study aimed to investigate whether nano-sized photocatalytic TiO_2_ possess adjuvant effect and promote allergic sensitization when administered in combination with OVA in mice.

## Materials and Methods

### Animals

Inbred female BALB/cJ mice aged 5–6 weeks were purchased from Taconic M&B, Ry, Denmark, and were housed as described previously [26]. Treatment of the animals followed procedures approved by The Animal Experiment Inspectorate, Denmark.

### Chemicals

Chicken egg OVA (CAS 9006-59-1) was grade V (purity ≥ 98%) from Sigma-Aldrich, St. Louis, MO, USA. The Al(OH)_3_ adjuvant was from Alhydrogel, Brenntag Biosector, Frederikssund, Denmark.

The TiO_2_ was VP Disp. W 2730 X from Degussa AG (Frankfurt am Main, Germany), now Evonik Industries, which is a 30 wt% dispersion of fumed TiO_2_. The hydrodynamic particle number size distribution of the TiO_2_ particles were determined by photon correlation spectroscopy using a Dynamic Laser Scatter (DLS) Zetasizer nano ZS (Malvern Inc., Malvern, UK) equipped with a 4 mW, 633 nm He-Ne Class I Laser. The results were calculated using the Dispersion Technology Software (DTS) *versus* 5.0 (Malvern Instruments Ltd.). TiO_2_ was suspended in 0.45 μm MilliQ filtered water, treated with ultrasound and measured in 1 ml disposable polystyrene cuvettes. For calculations, we used the refractive (*R*_i_) and absorption indices (*R*_s_) for rutile TiO_2_ (*R*_i_ = 2.903; *R*_s_ = 0.100) and standard properties for H_2_O. The density of TiO_2_ was set to 4.25 g/cm^3^.

### Immunization and airway challenge procedures

Mice (n = 7–8 per group) were primarily immunized on day 0 with 1 μg OVA alone or with 1 μg OVA in combination with either 2, 10, 50 or 250 μg TiO_2_ intraperitoneal (i.p.). A group receiving 1 μg OVA in combination with 270 μg Al(OH)_3_ served as positive adjuvant control.

**Fig. 1:**
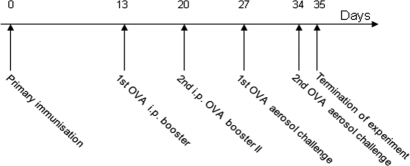
The study protocol. For details, confer the Materials and Methods section.

On days 13 and 20, all mice received a booster injection containing 0.1 μg OVA i.p. (fig. 1).

To avoid further particle agglomeration, the hydrous TiO_2_ suspension was diluted in sterile, pyrogen-free water as vehicle. Pyrogen-free saline was used for booster injections where OVA was administered without particles.

On days 27 and 34, all mice were challenged 20 min. with an aerosol of 1% OVA solution in saline.

The experiment was terminated on day 35. Blood samples were collected by heart puncture followed by bronchoalveolar lavage (BAL) as previously described [26].

Sera were assayed for content of OVA-specific IgE, IgG1 and IgG2a. The measurement of IgE was performed using a commercial IgE ELISA kit (OVA-IgE96 MD Biosciences, St Paul, MN, USA) according to manufacturer’s description. IgG1 and IgG2a were assayed as described previously [27]. Levels of interleukin (IL)-4, IL-5, IL-10 and interferon (IFN)-γ in BAL fluids were assayed by ELISA kits (eBioscience, San Diego, CA, USA) according to manufacturer’s description.

### Statistics

Antibody levels, numbers of inflammatory cells and levels of cytokines in BAL fluid in the four TiO_2_ groups were compared to the OVA control group by the Kruskal–Wallis test. If a statistically significant difference was apparent, the individual TiO_2_ groups were further compared to the OVA control group by the Mann-Whitney’s U-test. A *p*-value of less than 0.05 was considered statistically significant. Calculations were performed using the Minitab Statistical Software, Release 14 Xtra (Minitab Inc., State College, PA, USA).

## Results

### Characterization of particles

**Fig. 2:**
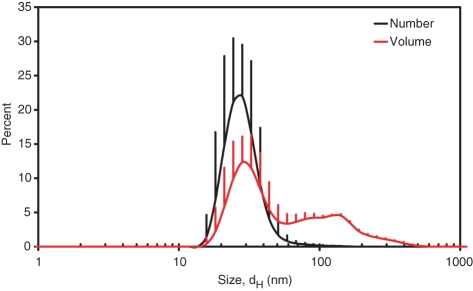
The hydrodynamic number and volume size distribution of the photocatalytic TiO_2_ (0.05 mg/ml) suspended in MilliQ-filtered water.

Dynamic Laser Scatter analysis showed that the size spectrum of the ultrasound-treated TiO_2_ particles suspended in MilliQ-filtered water was highly dominated by nano-sized particles (fig. 2). In the number size distribution, showed a single modal peak occurred around 28 nm. Plotted by volume, a polymodal size distribution with coarser submicron particles was resolved giving an average zeta-size of 140.04 ± 1.36 nm (Polydispersivety Index: 0.22). However, even by volume, more than 50% of the particles were smaller than 100 nm. This is in agreement with the manufacturer’s specification claiming that the mean aggregate size (d-50) was below 100 nm.

The surface area of the particles was calculated to be 34.1 m^2^/g.

### OVA-specific antibodies in serum

**Fig. 3:**
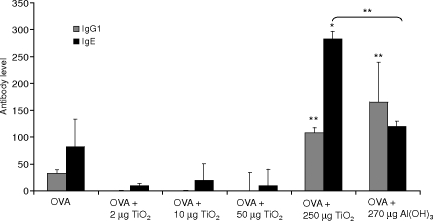
Levels of OVA-specific antibodies in serum. The antibody level is expressed as arbitrary concentration units for IgG1. For IgE the concentration unit is μg/10 ml. Data are median values with 75th percentile of 7–8 mice. Statistically increased level of antibody when compared to OVA control is shown as *p< 0.05 or **p< 0.01.

The IgE and IgG1 levels in the three lower doses of TiO_2_ were not statistically different from the OVA control group. The highest dose of TiO_2_, 250 μg, gave rise to significantly increased levels of IgE and IgG1 compared to the OVA control group (fig. 3). The Al(OH)_3_ adjuvant group gave rise to a significantly higher level of IgG1, whereas the increase in IgE was not statistically significant compared to the OVA control group. Furthermore, the level of IgE, but not IgG1 was significantly higher in the 250 μg TiO_2_ when compared to the Al(OH)_3_ group (*p* = 0.009).

Levels of IgG2a were below the limit of detection in all samples (data not shown).

### Inflammatory cells in BAL

**Fig. 4:**
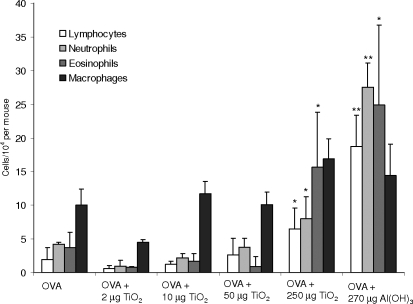
Number of inflammatory cells in bronchoalveolar lavage fluid. Data are median values with 75th percentile of 7–8 mice. Statistically increased cell number when compared to OVA control is shown as *p< 0.05 or **p< 0.01.

A significantly increased level of eosinophils, neutrophils and lymphocytes were seen in both the 250 μg TiO_2_ group and the adjuvant control group when compared to the OVA control group (confer fig. 4). Comparing the responses in the adjuvant control group and the 250 μg TiO_2_ groups revealed that the number of neutrophils were significantly higher in the adjuvant control group, whereas no difference was seen on the numbers of eosinophils, lymphocytes and macrophages.

### Cytokines in BAL supernatant

**Fig. 5:**
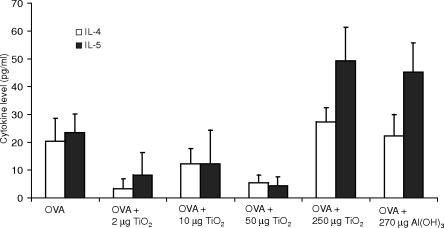
Levels of IL-4 and IL-5 in bronchoalveolar lavage fluid. Data are mean values with S.E.M. of 7–8 mice.

The Kruskal-Wallis analyses revealed a significant difference among groups in the levels of both the IL-4 (*p* = 0.032) and IL-5 (*p* = 0.002) (confer fig. 5); the highest levels of IL-4 and IL-5 were seen in the 250 μg TiO_2_ group and Al(OH)_3_ group. However, the pairwise comparison of exposure groups with the OVA control groups revealed no significant differences, i.e. none of the individual exposure groups differed from the OVA control group.

IL-10 and IFN-γ were below the limits of detection in all BAL samples (data not shown).

## Discussion

Our study showed that the photocatalytic TiO_2_ nanoparticles delivered as suspended in water possess adjuvant effect when administered in combination with OVA in mice. The adjuvant effect was suggested from the IgE and IgG1 levels, i.e. TiO_2_ primes a Th2-dominant immune response. Photocatalytic TiO_2_ nanoparticles were significantly more potent than Al(OH)_3_ to stimulate the IgE production, whereas the two compounds gave rise to an approximately equal increase in production of IgG1. Furthermore, TiO_2_ increased the influx of inflammatory cells in the lung. The increased level of eosinophils further supports that TiO_2_ stimulates an allergy-related immune response. Administration of 250 μg TiO_2_ gave rise to a 5-fold increase in the level of eosinophils, a response comparable to that induced by 270 μg Al(OH)_3_. As IgE is not significantly increased in the Al(OH)_3_ group, it could be speculated that the eosinophilic inflammation observed in this group is, at least in part, driven by the IgG1 antibody, which can be anaphylactic in the mouse after exposure to high allergen levels [28,29].

The mechanisms behind the adjuvant effect of particles have not been fully elucidated, but studies have shown that the size of a particle is important for its adjuvant activity. In general, adjuvanticity increases with decreasing particle size [21,25]. Due to the higher specific surface area of nanoparticles as compared to micron-sized particles, more antigen can be adsorbed to the same amount of particles [25]. Thus, nano (below 30 nm in diameter) TiO_2_ particles were found to bind more OVA per mass unit than fine (over 200 nm in diameter) TiO_2_ particles [21]. This may lead to the formation of an antigen depot with a prolonged release of antigen; a process that may increase antigenicity. Adsorption of antigen to a particle may also *per se* increase the antigenicity, as the most important professional antigen presenting cell type, the dendritic cells, are most effectively stimulated by antigen in particulate form [30]. Another proposal for the adjuvant mechanism is that ultra fine TiO_2_ may induce oxidative damage and inflammation; processes that increase the level of activity of immune competent cells. This is supported by an unpublished study conducted at our institute (Dr. A.T. Saber) showing that a single intratracheal instillation of 54 μg photocatalytic TiO_2_ nanoparticles induced acute neutrophilic inflammation in mice.

In conclusion, although micrometer-sized TiO_2_ particles in general are considered to be biologically relatively inert, this study has shown that TiO_2_ nanoparticles, which is a component in e.g. self-cleaning products, has adjuvant effect in mice. Thus, for TiO_2_, as shown for other substances, the toxicological risk assessments based on fine particles may not simply be extended to nanoparticles.

## References

[1] Nightingale JA, Maggs R, Cullinan P, Donnelly LE, Rogers DF, Kinnersley R (2000). Airway inflammation after controlled exposure to diesel exhaust particulates. Am J Respir Crit Care Med.

[2] Schaumann F, Borm PJ, Herbrich A, Knoch J, Pitz M, Schins RP (2004). Metal-rich ambient particles (particulate matter 2.5) cause airway inflammation in healthy subjects. Am J Respir Crit Care Med.

[3] D’Amato G (2002). Environmental urban factors (air pollution and allergens) and the rising trends in allergic respiratory diseases. Allergy.

[4] Peterson B, Saxon A (1996). Global increases in allergic respiratory disease: the possible role of diesel exhaust particles. Ann Allergy Asthma Immunol.

[5] Diaz-Sanchez D, Garcia MP, Wang M, Jyrala M, Saxon A (1999). Nasal challenge with diesel exhaust particles can induce sensitization to a neoallergen in the human mucosa. J Allergy Clin Immunol.

[6] Pandya RJ, Solomon G, Kinner A, Balmes JR (2002). Diesel exhaust and asthma: hypotheses and molecular mechanisms of action. Environ Health Perspect.

[7] Yanagisawa R, Takano H, Inoue KI, Ichinose T, Sadakane K, Yoshino S (2006). Components of diesel exhaust particles differentially affect Th1/Th2 response in a murine model of allergic airway inflammation. Clin Exp Allergy.

[8] Mortimer KM, Neas LM, Dockery DW, Redline S, Tager IB (2002). The effect of air pollution on inner-city children with asthma. Eur Respir J.

[9] Li N, Hao M, Phalen RF, Hinds WC, Nel AE (2003). Particulate air pollutants and asthma. A paradigm for the role of oxidative stress in PM-induced adverse health effects. Clin Immunol.

[10] Mauderly JL (2001). Diesel emissions: is more health research still needed?. Toxicol Sci.

[11] Nygaard UC, Hansen JS, Samuelsen M, Alberg T, Marioara CD, Lovik M (2009). Single-walled and multi-walled carbon nanotubes promote allergic immune responses in mice. Toxicol Sci.

[12] Inoue K, Takano H, Yanagisawa R, Koike E, Shimada A (2009). Size effects of latex nanomaterials on lung inflammation in mice. Toxicol Appl Pharmacol.

[13] Inoue K, Koike E, Yanagisawa R, Hirano S, Nishikawa M, Takano H (2009). Effects of multi-walled carbon nanotubes on a murine allergic airway inflammation model. Toxicol Appl Pharmacol.

[14] Ryman-Rasmussen JP, Tewksbury EW, Moss OR, Cesta MF, Wong BA, Bonner JC (2009). Inhaled multiwalled carbon nanotubes potentiate airway fibrosis in murine allergic asthma. Am J Respir Cell Mol Biol.

[15] Bernard BK, Osheroff MR, Hofmann A, Mennear JH (1990). Toxicology and carcinogenesis studies of dietary titanium dioxide-coated mica in male and female Fischer 344 rats. J Toxicol Environ Health.

[16] Chen JL, Fayerweather WE (1988). Epidemiologic study of workers exposed to titanium dioxide. J Occup Med.

[17] Lindenschmidt RC, Driscoll KE, Perkins MA, Higgins JM, Maurer JK, Belfiore KA (1990). The comparison of a fibrogenic and two nonfibrogenic dusts by bronchoalveolar lavage. Toxicol Appl Pharmacol.

[18] Gelis C, Girard S, Mavon A, Delverdier M, Paillous N, Vicendo P (2003). Assessment of the skin photoprotective capacities of an organo-mineral broad-spectrum sunblock on two ex vivo skin models. Photodermatol Photoimmunol Photomed.

[19] Sahu KK, Alex TC, Mishra D, Agrawal A (2006). An overview on the production of pigment grade titania from titania-rich slag. Waste Manag Res.

[20] Lomer MC, Thompson RP, Powell JJ (2002). Fine and ultrafine particles of the diet: influence on the mucosal immune response and association with Crohn’s disease. Proc Nutr Soc.

[21] de Haar C, Hassing I, Bol M, Bleumink R, Pieters R (2006). Ultrafine but not fine particulate matter causes airway inflammation and allergic airway sensitization to co-administered antigen in mice. Clin Exp Allergy.

[22] Renwick LC, Brown D, Clouter A, Donaldson K (2004). Increased inflammation and altered macrophage chemotactic responses caused by two ultrafine particle types. Occup Environ Med.

[23] Gurr JR, Wang AS, Chen CH, Jan KY (2005). Ultrafine titanium dioxide particles in the absence of photoactivation can induce oxidative damage to human bronchial epithelial cells. Toxicology.

[24] Granum B, Gaarder PI, Groeng E, Leikvold R, Namork E, Lovik M (2001). Fine particles of widely different composition have an adjuvant effect on the production of allergen-specific antibodies. Toxicol Lett.

[25] Nygaard UC, Samuelsen M, Aase A, Lovik M (2004). The capacity of particles to increase allergic sensitization is predicted by particle number and surface area, not by particle mass. Toxicol Sci.

[26] Larsen ST, Hansen JS, Hansen EW, Clausen PA, Nielsen GD (2007). Airway inflammation and adjuvant effect after repeated airborne exposures to di-(2-ethylhexyl)phthalate and ovalbumin in BALB/c mice. Toxicology.

[27] Larsen ST, Lund RM, Thygesen P, Poulsen OM, Nielsen GD (2003). Investigation of the adjuvant and immuno-suppressive effects of benzyl butyl phthalate, phthalic acid and benzyl alcohol in a murine injection model. Food Chem Toxicol.

[28] Oshiba A, Hamelmann E, Takeda K, Bradley KL, Loader JE, Larsen GL (1996). Passive transfer of immediate hypersensitivity and airway hyperresponsiveness by allergen-specific immunoglobulin (Ig) E and IgG1 in mice. J Clin Invest.

[29] Finkelman FD, Rothenberg ME, Brandt EB, Morris SC, Strait RT (2005). Molecular mechanisms of anaphylaxis: lessons from studies with murine models. J Allergy Clin Immunol.

[30] Chan RC, Wang M, Li N, Yanagawa Y, Onoe K, Lee JJ (2006). Pro-oxidative diesel exhaust particle chemicals inhibit LPS-induced dendritic cell responses involved in T-helper differentiation. J Allergy Clin Immunol.

